# Association of the ratio of visceral-to-subcutaneous fat volume with renal function among patients with primary aldosteronism

**DOI:** 10.1038/s41440-021-00719-w

**Published:** 2021-08-06

**Authors:** Tatsuya Haze, Moe Hatakeyama, Shiro Komiya, Rina Kawano, Yuki Ohki, Shota Suzuki, Yusuke Kobayashi, Akira Fujiwara, Sanae Saka, Kouichi Tamura, Nobuhito Hirawa

**Affiliations:** 1 Department of Medical Science and Cardiorenal Medicine, Yokohama City University Graduate School of Medicine, Yokohama, Japan; 2 Department of Nephrology and Hypertension, Yokohama City University Medical Center, Yokohama, Japan; 3 Center for Nobel and Exploratory Clinical Trials (Y-NEXT), Yokohama City University, Yokohama, Japan

**Keywords:** Computed tomography, Estimated glomerular filtration rate, Primary aldosteronism, Subcutaneous fat tissue, Visceral fat tissue

## Abstract

Patients with primary aldosteronism have a higher risk of chronic kidney disease. Visceral fat tissue is hypothesized to stimulate the adrenal glands to overproduce aldosterone, and aldosterone promotes visceral fat tissue to produce inflammatory cytokines. However, it is unclear whether the volume of accumulated visceral fat tissue is associated with renal impairment among patients with hyperaldosteronism. We conducted a single-center cross-sectional study to assess the association between the estimated glomerular filtration rate and the ratio of the visceral-to-subcutaneous fat volume calculated by computed tomography. One hundred eighty patients with primary aldosteronism were enrolled. The mean ± SD age was 52.7 ± 11.0 years, and 60.0% were women. The ratio of visceral-to-subcutaneous fat volume was highly correlated with the estimated glomerular filtration rate (*r* = 0.49, *p* < 0.001). In multiple linear regression models, the ratio of visceral-to-subcutaneous fat tissue volume was significantly associated with the estimated glomerular filtration rate (estimates: −4.56 mL/min/1.73 m² per 1-SD), and there was an interaction effect between the plasma aldosterone concentration and the ratio of visceral-to-subcutaneous fat volume (*p* < 0.05). The group with a higher plasma aldosterone concentration exhibited a steeper decline in eGFR than the lower plasma aldosterone concentration group when the ratio increased. The ratio of visceral-to-subcutaneous fat tissue volume was an independent risk factor for renal dysfunction. This association increased in the presence of a high plasma aldosterone concentration. Clinicians should pay attention to the ratio of visceral-to-subcutaneous fat tissue volume and encourage primary aldosteronism patients to improve their lifestyle in addition to treating renin-aldosterone activity.

## Abstract (web summary)

The ratio of visceral-to-subcutaneous fat tissue volume was significantly associated with eGFR among 180 primary aldosteronism patients, showing an interaction between the ratio and plasma aldosterone concentration.

## Graphical abstract

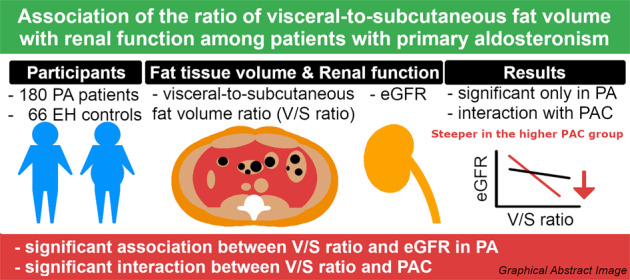

## Introduction

Primary aldosteronism (PA) is caused by aldosterone overproduction from the adrenal glands and is the most common cause of secondary hypertension, with an estimated prevalence of 5–20% in hypertensive patients [1, 2]. Aldosterone is involved in inflammatory and fibrotic processes within the kidney, as well as in mesangial cell proliferation and podocyte injury [3, 4], leading to perivascular fibrosis, glomerulosclerosis, and interstitial fibrosis [4]. Thus, patients with PA have an increased risk of kidney diseases compared with patients with essential hypertension (EH) [5]. Adrenalectomy or mineralocorticoid receptor antagonist (MRA) treatment is recommended for patients with PA [2]. However, patients with PA treated by MRA exhibit a faster decline in renal function than those with EH [6]. It is important to clarify the risk factors for renal failure in patients with PA and identify methods to improve their renal prognosis.

Visceral fat (VF) tissue has been proposed as an independent risk factor for hypertension and chronic kidney disease in non-PA populations [7, 8]. The ratio of visceral-to-subcutaneous fat tissue area at the umbilical level is a known metric of adipose tissue dysfunction [9] and has been reported to be a risk factor for end-stage renal disease in diabetic patients [10]. On the other hand, VF tissue is deeply involved in the pathology of PA. VF tissue is hypothesized to stimulate the adrenal glands to overproduce aldosterone, which promotes VF tissue to produce inflammatory cytokines [11–13]. This suggests that VF accumulation becomes more harmful under the condition of higher mineralocorticoid receptor activity.

However, whether VF accumulation is a significant risk factor for renal impairment in PA patients and precisely how VF accumulation and renin-aldosterone activity interact in these patients remains unknown. Therefore, we assessed whether the ratio of visceral-to-subcutaneous fat tissue is associated with renal function in patients with PA to examine whether the control of VF accumulation may improve the renal prognosis of PA.

## Methods

### Participants

This was a single-center cross-sectional observational study. We enrolled patients with PA or EH aged over 20 who underwent a confirmatory test (i.e., captopril challenge test, furosemide-upright test, or saline infusion test) between January 2006 and April 2021 at our division in Yokohama City University Medical Center (Yokohama, Japan). PA or EH was diagnosed based on the guidelines of the Japan Endocrine Society [2] and the Japanese Society of Hypertension [14].

For the analyses, we selected individuals (1) who were diagnosed with PA or EH, (2) who had not been treated by adrenalectomy or MRA, and (3) whose records included an assessment of the estimated glomerular filtration rate (eGFR) and computed tomography (CT) findings. We excluded patients who underwent renal replacement therapy. Finally, 180 patients with PA (the PA group) and 66 patients with EH (the EH group) were included (Supplementary Fig. 1). We investigated the PA group as the primary analysis. We then performed an additional analysis in the EH group to check whether the findings were generalizable to our patients with EH.

### Tests for primary aldosteronism

To determine whether aldosterone overproduction was lateralized in a unilateral adrenal gland, we performed adrenal venous sampling (AVS) in the PA group [2, 14]. A total of 119 patients underwent AVS, and the sampling was successful in 104. Among these 104 patients, 32 were suspected of having lateralized overproduction. For details, see the online Supplemental Material (eMethods) [15–17].

### Renal function

Serum creatinine was assayed using an enzymatic method, and eGFR was calculated using the Chronic Kidney Disease Epidemiology Collaboration (CKD-EPI) equation for Japanese individuals [18]. The eGFR was measured every two months from 6 months before the diagnosis to the date of the diagnosis (i.e., at month-6, month-4, month-2, and month 0) (see eMethods). Baseline eGFR was calculated as the mean of all measured eGFR values for each individual. Quantitative urinalysis was performed using spot urine specimens. Urinary protein was calculated from the protein-to-creatinine ratios. Urinalysis data were collected at the time of diagnosis.

### Computed tomography

All patients underwent multi-slice helical CT with a 0.5–5-mm slice thickness at the time of diagnosis. The volume and area of the VF and subcutaneous fat (SF) tissue were automatically estimated using volume analyzer software (SYNAPSE VINCENT®, Fujifilm Medical Co., Tokyo). The total volume of the psoas muscle was also detected by the software. The VF area (VFA), SF area (SFA), and weight circumference (WC) were calculated at the mid-umbilical level. The VF volume (VFV) and SF volume (SFV) were calculated at the level from the pelvic floor to the diaphragm (see eMethods).

### Other measurements

The plasma aldosterone concentration (PAC) and plasma renin activity (PRA) were measured by radioimmunoassay methods with commercially available kits. The data were collected retrospectively from the patients’ medical records. The clinical laboratory data were collected under fasting conditions. For details, see eMethods [14, 19, 20].

### Ethics

This study was conducted according to the guidelines of the Declaration of Helsinki and the guidelines for clinical studies published by the Ministry of Health and Labor, Japan, and was approved by the ethics committee of our hospital. Informed consent was received from each contactable patient, and if they were not contactable (e.g., dead or missing), we used an opt-out method.

### Statistical methods

Assuming that missing data for covariates occurred independently of missing measurements of eGFR, all variables with missing data (Supplementary Table 1) were imputed with 100 data sets using chained equations [21].

All analyses were performed with R version 4.0.4 using the default strings of the mice 3.13.0 package for imputation. Significance was defined as *p* < 0.05 using 2-sided tests.

### Primary analyses in the PA group

First, we conducted correlation tests and linear regression models to investigate the relationship between the levels of fat tissue accumulation and the renal function in the PA group.

### Correlation test

We tested the correlation between the levels of fat accumulation (i.e., VFV, SFV, VFA, SFA, the ratio of VFV to SFV [the VFV/SFV ratio], and the ratio of VFA to SFA [the VFA/SFA ratio]) and the levels of renin-aldosterone activity (i.e., PAC, PRA, or ARR) or the renal parameters (i.e., eGFR or urinary protein). We calculated Pearson’s product-moment correlation coefficients. We confirmed the normality assumption via graphics using histograms and normal quantile-quantile plots. PAC, PRA, ARR, and urinary protein were log-transformed because of their skewed distribution. We also tested for total fat volume (=VFV + SFV), total fat area (=VFA + SFA), WC, and body mass index (BMI). Finally, we divided the PA group without missing values for urinary protein into two subgroups according to the presence or not of urinary protein (i.e., 0.15 mg per gram of creatinine) [22].

### Linear regression model

We used multiple linear regression models to calculate the estimates and 95% confidence intervals (CIs) for the association of eGFR with VFV, SFV, VFA, SFA, the VFV/SFV ratio, or the VFA/SFA ratio. Possible violations of these assumptions were examined by visually inspecting scatter plots of the residuals and predicted values. Model 1 was unadjusted. Model 2 was adjusted for age, sex, BMI, and the laterality of aldosterone hypersecretion by the adrenal glands (unilateral vs. bilateral or undeterminable). Model 3 was adjusted for the covariates included in Model 2 and a history of diabetes, the prevalence of dyslipidemia, current smoking status, and mean artery pressure. Model 4 was adjusted for the covariates included in Model 3 and serum potassium, log-transformed duration of hypertension, log-transformed urinary protein, and log-transformed PAC. Exposures were included separately in the models. Covariates were selected a priori [23, 24].

### Interaction

We assessed the heterogeneity in the association between exposures and outcomes by PAC with the inclusion of multiplicative interaction terms. We divided the PA group into two subgroups according to the median PAC and performed subgroup analyses. We also tested the interactions for sex, findings of adrenal glands on CT, and laterality in AVS using multiplicative interaction terms.

### Sensitivity analyses

We conducted eleven sensitivity analyses, including using VFV per body weight (VFV/BW) and SFV per body weight (SFV/BW), subgroups dividing participants by laterality in AVS, or performing complete-case analyses without imputation methods (see eMethods).

### Subgroup analyses by sex

Assuming that VF and SF tissue accumulations were strongly associated with the sex [25], we performed subgroup analyses comparing women and men (see eMethods).

### Secondary analysis in the EH group

As a secondary analysis, we conducted correlation tests in the EH group (*n* = 66) to investigate whether the relationship between the VFV/SFV ratio and eGFR was generalizable to patients with EH.

## Results

The final analytic sample for the primary analyses included 180 patients with PA (the mean ± SD of age was 52.7 ± 11.0 years, BMI was 25.1 ± 4.7 kg/m^2^, and 60.0% were female). The clinical characteristics of the PA group are shown in Table 1. The mean ± SD values of the VFV/SFV ratio and the VFA/SFA ratio were 0.8 ± 0.4 and 0.8 ± 0.6, respectively.

**Table 1:** Characteristics of participants with PA

Characteristics	PA group (*n* = 180)	The higher PAC group (*n* = 90)	The lower PAC group (*n* = 90)	*p*-value
Age, years	52.7 ± 11.0	52.0 ± 11.6	53.4 ± 10.4	0.38
Women, *n* (%)	108 (60.0)	51 (56.7)	57 (63.3)	0.36
BMI, kg/m^2^	25.1 ± 4.7	25.6 ± 4.3	24.5 ± 4.9	0.10
History of diabetes, *n* (%)	18 (10.0)	9 (10.0)	9 (10.0)	1.00
Current smokers, *n* (%)	34 (18.9)	13 (14.4)	21 (23.3)	0.13
History of cardiovascular disease, *n* (%)	11 (6.1)	7 (7.8)	4 (4.4)	0.35
Known duration of hypertension, years	3.6 (1.2, 9.0)	5.0 (2.0, 10.8)	2.4 (0.7, 5.4)	<0.001^***^
Antihypertensive medication use, *n* (%)	71 (39.4)	40 (44.4)	31 (34.4)	0.17
Calcium-channel blockers, *n* (%)	68 (37.8)	38 (42.2)	30 (33.3)	0.22
Angiotensin-converting enzyme inhibitors or Angiotensin receptor blockers, *n* (%)	10 (5.6)	6 (6.7)	4 (4.4)	0.52
Thiazides, *n* (%)	4 (2.2)	2 (2.2)	2 (2.2)	1.00
Alpha-blockers, *n* (%)	12 (6.7)	10 (11.1)	2 (2.2)	<0.05^*^
Beta-blockers, *n* (%)	3 (1.7)	2 (2.2)	1 (1.1)	0.56
SBP, mmHg	144 ± 16	145 ± 15	143 ± 16	0.30
DBP, mmHg	90 ± 10	90 ± 10	91 ± 10	0.41
PAC, pg/mL	175.0 (129.0, 234.6)	234.8 (195.8, 304.5)	129.0 (99.7, 150.9)	<0.001^***^
PRA, ng/mL per hour	0.4 (0.2, 0.6)	0.5 (0.3, 0.8)	0.3 (0.2, 0.5)	<0.01^**^
ARR (=PAC/PRA)	432.5 (294.9, 670.7)	499.6 (322.5, 1118.4)	359.7 (263.7, 503.3)	<0.001^***^
eGFR, mL/min/1.73 m^2^	77.5 ± 14.8	76.4 ± 17.5	78.7 ± 11.7	0.29
Urinary protein, mg/gCr	97.7 (59.0, 278.9)	115.8 (61.8, 662.9)	91.0 (57.2, 154.4)	0.06
Serum potassium, mEq/L	3.9 ± 0.4	3.8 ± 0.4	4.0 ± 0.3	<0.05^*^
Uric acid, mg/dL	5.4 ± 1.3	5.4 ± 1.3	5.3 ± 1.3	0.51
HbA1c, %	5.6 (5.3, 6.2)	5.6 (5.4, 6.2)	5.6 (5.3, 6.2)	1.00
Total cholesterol, mg/dL	206.6 ± 40.3	207.5 ± 40.2	205.7 ± 40.2	0.87
Triglycerides, mg/dL	115.7 (79.9, 185.2)	129.4 (88.9, 206.5)	101.4 (76.4, 162.2)	0.06
LDL cholesterol, mg/dL	119.3 ± 29.0	120.5 ± 30.2	118.1;± 27.8	0.60
HDL cholesterol, mg/dL	59.6 ± 17.2	58.7 ± 16.4	60.4 ± 17.9	0.53
WC, cm	88.0 ± 10.6	88.5 ± 10.4	87.4 ± 10.9	0.51
VFA, cm^2^	118.3 ± 65.9	123.2 ± 67.3	113.3 ± 64.4	0.31
SFA, cm^2^	171.4 ± 84.0	171.2 ± 84.3	171.6 ± 84.1	0.97
VFA/SFA ratio	0.8 ± 0.6	0.9 ± 0.8	0.7 ± 0.4	0.13
VFV, cm^3^	3139.2 ± 1884.9	3280.7 ± 2036.4	2997.8 ± 1719.8	0.32
SFV, cm^3^	4484.6 ± 2245.4	4470.9 ± 2303.8	4498.3 ± 2198.3	0.94
VFV/SFV ratio	0.8 ± 0.4	0.8 ± 0.5	0.7 ± 0.4	0.32
Total psoas muscle volume, cm^3^	304.7 ± 113.0	310.0 ± 101.1	299.5 ± 124.1	0.53

Data are expressed as the mean±SD for unskewed variables and median (interquartile range) for skewed variables. Comparison tests were performed between two subgroups with the Student’s t-test, Wilcoxon rank-sum test, or chi-square test where appropriate

*ARR* aldosterone-to-renin ratio, *BMI* body mass index, *DBP* diastolic blood pressure, *eGFR* estimated glomerular filtration rate, *gCr* per gram of creatinine, *HbA1c* hemoglobin A1c, *HDL* high-density lipoprotein, *LDL* low-density lipoprotein, *PA* primary aldosteronism, *PAC* plasma aldosterone concentration, *PRA* plasma renin activity, *SBP* systolic blood pressure, *SFA* subcutaneous fat area, *SFV* subcutaneous fat volume, *VFA* visceral fat area, *VFV* visceral fat volume, *WC* waist circumference

*p < 0.05; **p < 0.01; ***p < 0.001

### Correlation between abdominal fat accumulation and renin-aldosterone activity or renal function among patients with PA

The estimated Pearson’s product-moment correlation coefficients (*r*-values) between abdominal fat accumulation and renin-aldosterone activity or renal function are shown in Table 2. Both VF and SF accumulation parameters exhibited no significant correlation with PAC, PRA, or ARR. For renal parameters, VFV was inversely correlated with eGFR. The VFV/SFV ratio was highly correlated with eGFR (*r* [95% CIs]: −0.49 [−0.60, −0.37]), and this correlation was significantly stronger than that between VFV alone and eGFR (*p* < 0.01). The results for VFA and SFA were similar to those for VFV and SFV. Total abdominal fat volume, total fat area, WC, and BMI were not correlated with PAC, PRA, ARR, eGFR, or urinary protein in this study (data not shown). When we used the VFV/BW and SFV/BW to adjust for physique, VFV/BW remained inversely correlated with eGFR (*r* [95% CIs]: −0.31 [−0.43, −0.17]), and SFV/BW exhibited a positive correlation with eGFR (*r* [95% CIs]: 0.15 [0.00, 0.29]).

**Table 2:** Correlation between abdominal fat accumulation and renin-aldosterone activity or renal function among patients with PA (*n* = 180)

Estimated Pearson’s product-moment correlation coefficients (*r*)						
Renin-aldosterone activity						
	VFV, cm³	SFV, cm³	VFV/SFV ratio	VFA, cm^2^	SFA, cm^2^	VFA/SFA ratio
log PAC, pg/mL	0.02 (−0.13, 0.17)	−0.01 (−0.16, 0.13)	0.01 (−0.14, 0.15)	0.01 (−0.14, 0.15)	−0.00 (−0.15, 0.14)	0.01 (−0.14, 0.16)
log PRA, ng/mL/h	0.07 (−0.07, 0.22)	0.06 (−0.09, 0.20)	0.06 (−0.09, 0.20)	0.11 (−0.04, 0.25)	0.08 (−0.07, 0.22)	0.06 (−0.09, 0.20)
log ARR	−0.05 (−0.20, 0.09)	−0.06 (−0.21, 0.09)	−0.05 (−0.19, 0.10)	−0.09 (−0.24, 0.05)	−0.08 (−0.22, 0.07)	−0.05 (−0.19, 0.10)

Renal function						
	VFV, cm³	SFV, cm³	VFV/SFV ratio	VFA, cm^2^	SFA, cm^2^	VFA/SFA ratio
eGFR, mL/min/1.73 m^2^	−0.36*** (−0.48, −0.22)	0.10 (−0.05, 0.24)	−0.49*** (−0.60, −0.37)	−0.24*** (−0.38, −0.10)	0.10 (−0.05, 0.24)	−0.23** (−0.37, −0.09)
log Urinary protein, mg/gCr	0.13 (−0.10, 0.35)	0.08 (−0.10, 0.25)	0.01 (−0.19, 0.21)	0.06 (−0.15, 0.25)	0.05 (−0.12, 0.22)	0.04 (−0.14, 0.23)

Estimated Pearson’s product-moment correlation coefficients (95% confidence intervals) are shown

*ARR* aldosterone-to-renin ratio, *eGFR* estimated glomerular filtration rate, *gCr* per gram of creatinine, *PA* primary aldosteronism, *PAC* plasma aldosterone concentration, *PRA* plasma renin activity, *SFA* subcutaneous fat area, *SFV* subcutaneous fat volume, *VFA* visceral fat area, *VFV* visceral fat volume

**p < 0.01; ***p < 0.001

Urinary protein and the VFV/SFV ratio were not correlated in the overall PA group. However, when we divided the PA group without missing values for urinary protein by the presence of proteinuria, the VFV and the VFV/SFV ratio were significantly correlated with urinary protein levels in the participants with proteinuria (*r* [95% CIs]: 0.63 [0.46, 0.79] for the VFV/SFV ratio) (Supplementary Table 2).

### Linear association between abdominal fat accumulation and renal function among patients with PA

**Fig. 1:**
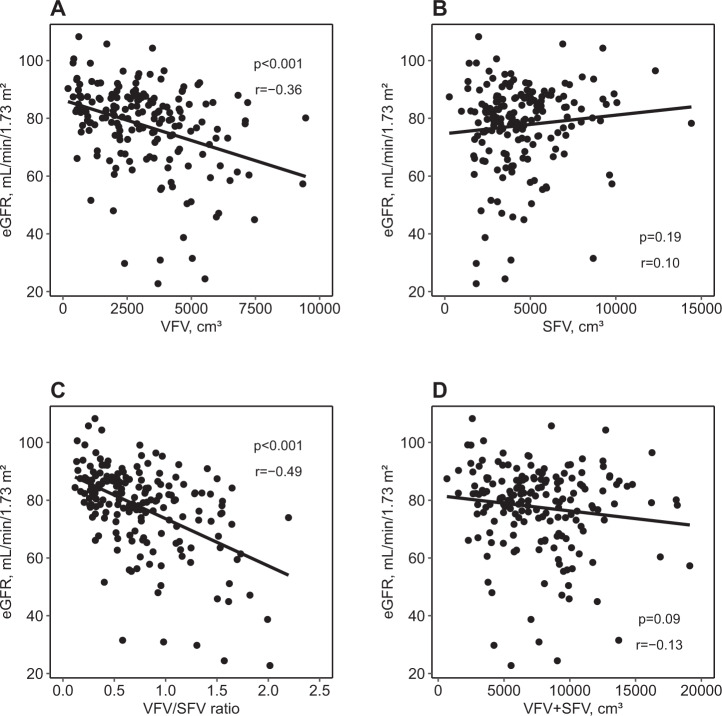
Scatter plots of the relationship between the visceral or subcutaneous fat volume and eGFR. Scatter plots of the relationship between eGFR and (**A**) VFV, (**B**) SFV, (**C**) the VFV/SFV ratio, and (**D**) the sum of VFV and SFV in the PA group are shown. Each black dot represents a value observed in an individual patient. The black lines represent simple linear regression models. The *p*-values were calculated for Pearson’s product-moment correlation coefficients (*r*-values). eGFR=estimated glomerular filtration rate; SFV subcutaneous fat volume; VFV visceral fat volume

Unadjusted and adjusted estimates for the association of eGFR with the levels of fat tissue accumulation in the linear regression models are shown in Table 3. In the unadjusted model (Model 1), VFV and the VFV/SFV ratio were significantly associated with eGFR (estimates [95% CIs]: VFV, −5.28 [−7.32, −3.24]; the VFV/SFV ratio, −7.33 [−9.23, −5.43] mL/min/1.73 m²). After multivariable adjustment for known risk factors, including PAC, the VFV/SFV ratio was still significantly associated with eGFR (estimates [95% CIs]: −4.56 [−6.98, −2.14] mL/min/1.73 m²). VFV, VFV/BW, and SFV/BW were significantly associated with eGFR in the unadjusted models but not in the adjusted models. Scatter plots of eGFR and VFV, SFV, the VFV/SFV ratio, and total fat volume are shown in Fig. 1.

**Table 3:** Association between abdominal fat accumulation and eGFR among patients with PA (*n* = 180)

Estimates for eGFR per each 1-SD higher						
	VFV, cm³	SFV, cm³	VFV/SFV ratio	VFA, cm^2^	SFA, cm^2^	VFA/SFA ratio
Model 1	−5.28*** (−7.32, −3.24)	1.44 (−0.73, 3.61)	−7.33*** (−9.23, −5.43)	−3.62** (−5.73, −1.50)	1.46 (−0.71, 3.63)	−3.45** (−5.57, −1.33)
Model 2	−2.48 (−5.15, 0.19)	2.58 (−0.35, 5.51)	−4.95*** (−7.57, −2.32)	0.90 (−1.52, 3.32)	2.61 (−0.21, 5.42)	−0.70 (−2.48, 1.08)
Model 3	−2.04 (−4.81, 0.73)	2.61 (−0.30, 5.53)	−4.84*** (−7.41, −2.27)	1.20 (−1.28, 3.68)	2.51 (−0.27, 5.29)	−0.36 (−2.12, 1.39)
Model 4	−2.11 (−4.76, 0.54)	2.60 (−0.23, 5.44)	−4.56*** (−6.98, −2.14)	0.38 (−2.07, 2.82)	2.42 (−0.25, 5.09)	−0.37 (−2.10, 1.36)

Unadjusted and adjusted estimates (95% confidence intervals) for eGFR associated with one-SD higher VFV, SFV, the VFV/SFV ratio, VFA, SFA, and the VFA/SFA ratio are shown. Each one-SD increment is as follows: VFV, 1884.9 cm³; SFV, 2245.4 cm³; VFV/SFV ratio, 0.4; VFA, 65.9 cm²; SFA, 84.0 cm²; and VFA/SFA ratio, 0.6. Model 1 was unadjusted. Model 2 was adjusted for age, sex, BMI, and laterality of aldosterone hypersecretion. Model 3 was adjusted for the covariates included in Model 2 and history of diabetes, prevalence of dyslipidemia, current smoking status, and mean artery pressure. Model 4 was adjusted for the covariates included in Model 3 and serum potassium, log-transformed duration of hypertension, log-transformed urinary protein, and log-transformed PAC. Exposures were included in models separately

*BMI* body mass index, *eGFR* estimated glomerular filtration rate, *PA* primary aldosteronism, *PAC* plasma aldosterone concentration, *SFA* subcutaneous fat area, *SFV* subcutaneous fat volume, *VFA* visceral fat area, *VFV* visceral fat volume

**p < 0.01; ***p < 0.001

### Effect of the interaction between the VFV/SFV ratio and PAC on eGFR among patients with PA

When we assessed the effect on eGFR of the interaction between the VFV/SFV ratio and PAC, the multiplicative interaction terms were significantly associated with eGFR (estimates [95% CIs]: −1.48 [−2.76, −0.20] mL/min/1.73 m², *p* < 0.05), whereas the simple main effects (i.e., estimates for the VFV/SFV ratio alone or PAC alone) were still significant (estimates [95% CIs]: −4.52 [−6.90, −2.13] mL/min/1.73 m² for the VFV/SFV ratio, −2.21 [−3.88, −0.53] mL/min/1.73 m² for log PAC). We also performed the regression without using log-transformation, and similar results were obtained (estimates [95% CIs]: −1.45 [−2.75, −0.16] mL/min/1.73 m² for the interaction, −3.80 [−6.09, −1.51] mL/min/1.73 m² for the VFV/SFV ratio, −3.55 [−5.20, −1.91] mL/min/1.73 m² per 1-SD higher [132.8 pg/mL] for PAC).

**Fig. 2:**
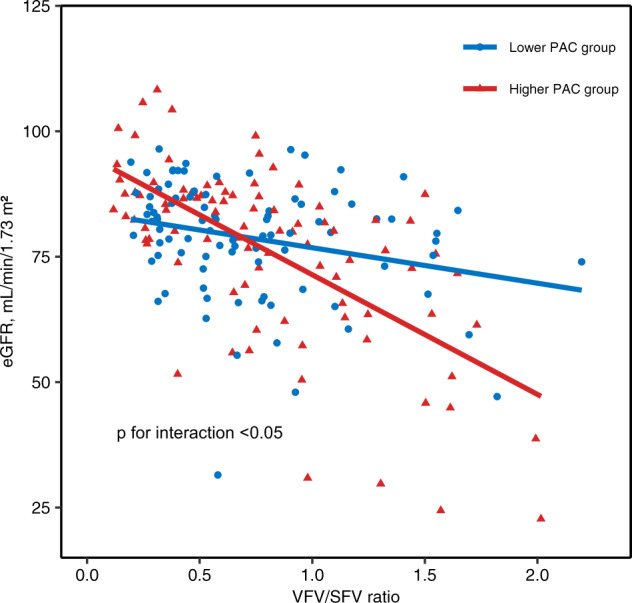
Interaction between PAC and the ratio of visceral-to-subcutaneous fat tissue volume. Scatter plots between eGFR and the VFV/SFV ratio in the PA group are shown. Each dot represents a value for each individual patient. Circular dots (red) show data for the group with higher PAC levels (PAC>175.0 pg/mL). Triangular dots (blue) show data for the group with lower PAC levels (PAC≤175.0 pg/mL). The red line represents the simple regression model for the higher PAC group. The blue line represents the simple regression model for the lower PAC group. The *p*-value was calculated for the multiple interaction terms between the VFV/SFV ratio and the higher PAC vs. lower PAC group in the regression model. eGFR estimated glomerular filtration rate, SFV subcutaneous fat volume, VFV visceral fat volume

In subgroup analyses between the lower PAC group (PAC ≤ 175.0 pg/mL) and the higher PAC group (PAC > 175.0 pg/mL), the VFV/SFV ratio was significantly associated with eGFR in both subgroups in the unadjusted model but was not significantly associated with eGFR in the adjusted models in the lower PAC group (Supplementary Table 3). The adjustment included the known duration of hypertension, which was longer in the higher PAC group. The characteristics of these subgroups are shown in Table 1. The correlation test revealed a significant correlation between the VFV/SFV ratio and eGFR in both subgroups (*r*-value [95% CIs]: −0.26 [−0.44, −0.06] for the lower PAC group, −0.64 [−0.75, −0.50] for the higher PAC group). The *r*-value in the higher PAC group was higher than that in the lower PAC group (*p* < 0.001). Scatter plots and the estimated linear regression models (unadjusted) in the two subgroups are shown in Fig. 2, demonstrating the steeper decline in the association between eGFR and the VFV/SFV ratio in the higher PAC group than in the lower PAC group (*p* < 0.05).

### Sensitivity analyses

We conducted eleven sensitivity analyses to confirm the validity of our primary analyses: (1) the results of linear regression were comparable when we used VFV/BW and SFV/BW instead of VFV and SFV. The VFV/SFV ratio was significantly associated with eGFR when we adjusted for any of the following in the linear regression models: (2) PRA (Supplementary Table 4, Model 5); (3) ARR (Supplementary Table 4, Model 6); (4) the use of angiotensin-converting enzyme inhibitors (ACEi) or angiotensin receptor blockers (ARB) (Supplementary Table 4, Model 7); (5) hemoglobin A1c, low-density lipoprotein cholesterol, high-density lipoprotein cholesterol, and triglycerides (data not shown); (6) the number of measurements of eGFR (data not shown); or (7) the total psoas muscle volume (Supplementary Table 4, Model 8). The results were also similar when we performed (8) subgroup analyses of participants with lateralization of the overproduction of aldosterone by the adrenal glands in AVS (*n* = 32) and participants without such lateralization (*n* = 72), (9) subgroup analyses of participants who had adenomatous adrenal glands on CT (*n* = 61) and those who did not (*n* = 119) (data not shown), or (10) subgroup analyses excluding participants who were taking ACEis, ARBs, diuretics, beta-blockers, or thiazolidines that have been reported to affect PAC or the VFV/SFV ratio (Supplementary Table 4, Model 9) [26, 27], or (11) complete-case analyses among participants without missing values (Supplementary Table 4, Model 10).

We assessed the heterogeneity among participants using interactive terms for the findings of adrenal glands on CT or indications of the lateralization of aldosterone production on AVS. However, these variables were not suggested to interact in the association between the VFV/SFV ratio and eGFR.

Although no evidence was found to suggest that sex interacted in the association between the VFV/SFV ratio and eGFR (*p* > 0.19 for interaction), there was a difference in the distribution of the VFV/SFV ratio between men and women (Supplementary Fig. 2). To investigate this point, we performed subgroup analyses by sex. For the details, see the online Supplemental Material (eMethods). Although the association between the VFV/SFV ratio and eGFR was similar and significant in correlation tests (*r*-value [95% CIs]: −0.45 [−0.59, −0.28], for the female group, −0.44 [−0.61, −0.23] for the male group, both *p* < 0.001) and in the linear regression models for both the male and female subgroups (Supplementary Table 5), the male group showed a significant interaction between the VFV/SFV ratio and PAC for eGFR (*p* < 0.05), whereas the female group did not show such an interaction (Supplementary Fig. 3).

### Secondary analyses: correlation between abdominal fat accumulation and renal function among patients with EH

The characteristics of the EH group (*n* = 66) are shown in Supplementary Table 6. The EH group showed significantly higher levels of PRA than the PA group. No evidence was found to suggest that there was a significant correlation between abdominal fat accumulation and renal function among patients with EH (all *p* > 0.26, Supplementary Table 7). Scatter plots of eGFR and the VFV/SFV ratio are shown in Supplementary Figure 4.

## Discussion

In this single-center cross-sectional observational study, the abdominal VFV/SFV ratio was significantly associated with renal function in patients with PA. When the VFV/SFV ratio increased by 0.4, the estimated eGFR decreased by −7.33 mL/min/1.73 m² in our unadjusted model. Although additional studies are required to confirm the causal relationship, our findings suggested the possibility that a reduction in the VFV/SFV ratio might improve renal function in PA patients independently of the known risk factors.

We found a strong correlation between VFV and eGFR. SFV had a slight but insignificant correlation, while a significant correlation was found when SFV was divided by body weight. VFV and SFV had inverse correlation coefficients, suggesting that the association of eGFR with VF tissue differs from that of eGFR with SF tissue. Fat tissue accumulation can lead to hyperfiltration in glomeruli and an increased eGFR [28]. However, the total fat tissue volume (VFV + SFV) was not correlated with eGFR in this study. Instead, the VFV/SFV ratio exhibited a stronger correlation with eGFR, suggesting that the balance of VFV and SFV, rather than the total amount of fat tissue, is important for renal function. After adjusting for the known risk factors, including the levels of renin-aldosterone activity (i.e., PAC, PRA, or ARR) [24, 29] with linear regression, only the VFV/SFV ratio demonstrated an independent association with eGFR. The VFV/SFV ratio may be a more informative indicator of renal function than VFV or SFV alone. Fat tissue accumulation and eGFR may be confounded by muscle volume. However, this association was still significant when we additionally adjusted for the total psoas muscle volume. The VFA and the VFA/SFA ratio were similarly correlated with eGFR; however, VFV and the VFV/SFV ratio may be better indicators. The positions of the internal organs and intestinal content may vary among individuals. The three-dimensional volume estimation of abdominal fat tissue is likely to be more helpful than two-dimensional estimation to assess the precise levels of fat tissue accumulation.

Fat tissue produces a diverse range of cytokines, which are both protective and harmful to the arteries and kidneys [30, 31]. Adiponectin is a protective adipokine suppressing arteriosclerosis [32, 33]. Conversely, leptin has been reported to increase sympathetic nerve activity in the kidneys [34], induce inflammation [35], and increase urinary protein [36]. Fat tissue accumulation leads to reduced production of adiponectin and increased production of leptin [37, 38]. The “lipid overflow ectopic fat model” suggests that surplus energy is primarily stored in SF tissue, and when this intrinsic metabolic sink becomes dysfunctional due to genetic polymorphism or extrinsic factors, such as cigarette use, energy can alternatively be deposited in visceral compartments [9]. Because VF tissue may have a higher capability of producing inflammatory cytokines than SF tissue [39–42], the accumulation of VF tissue may disturb the balance between benign and harmful adipokine secretion. SF accumulation also indicates that the energy surplus is within the limit and does not cause an overflow [9], suggesting that adipose tissue functions normally. Thus, the ratio of visceral-to-subcutaneous fat tissue is considered to be a potential index reflecting the dysfunctional state in adipose tissues and has been reported to be associated with increased risks of renal impairment [42] and mortality in a non-PA population [43]. Our study confirmed that the ratio of visceral-to-subcutaneous fat tissue is independently associated with renal impairment in patients with PA.

In addition, we revealed that an interaction between the levels of circulating aldosterone and the VFV/SFV ratio was involved in the decline in eGFR in PA patients. Aldosterone can affect adipocytes via mineralocorticoid receptors and regulate the differentiation of adipocytes [44] and adipogenesis [30], thereby increasing the harmful production of inflammatory adipokines such as leptin, interleukin-6 (IL-6), and tissue necrosis factor-α (TNF-α) and reducing the production of adiponectin [13, 45]. Conversely, several factors that stimulate the adrenal glands to produce aldosterone were found in adipocytes [11], and leptin is one such stimulant [12]. This suggested a pathological interaction between aldosterone and fat tissue accumulation;[30] however, it is unclear whether and to what extent this interaction is clinically relevant. We demonstrated a significant interaction between the VFV/SFV ratio and PAC levels in eGFR decline, and participants who had higher PAC levels exhibited a steeper decline in eGFR when their VFV/SFV ratio increased. Thus, there is a possibility that control of the VFV/SFV ratio may improve renal function, especially in patients with high aldosterone levels. Weight loss and thiazolidines have been reported to improve the ratio of visceral-to-subcutaneous fat tissue [27, 46]. These treatments may also improve renal dysfunction in PA patients. Clinicians may need to encourage PA patients to improve their lifestyle and reduce VF tissue accumulation in addition to treating renin-aldosterone activity.

In a previous report, VFA and PAC were correlated in patients with idiopathic hyperaldosteronism [47]. One report showed that VFA was significantly lower in an aldosterone-producing adenoma (APA) group than in patients with EH [48]. However, the association between the levels of renin-aldosterone activity and those of VF tissue accumulation remains controversial. A large population-based study reported that VFAs and SFAs were not associated with PAC in a general population [49]. Another study reported that PAC and VFA were correlated in normotensive women [50]. In our study, PAC, PRA, and ARR were not correlated with the levels of VF tissue accumulation. Assuming heterogeneity, we performed three subgroup analyses including sex, PA subtypes, and CT findings. No statistical evidence was found to suggest that these factors interacted in the association between the VFV/SFV ratio and eGFR. However, in the subgroups by sex, only the male group showed a significant interaction effect of PAC in the association between the VFV/SFV ratio and eGFR, while the female group did not. These findings imply that there is a difference in the relationships among the VFV/SFV ratio, PAC, and eGFR depending on sex. Estrogens promote SF deposition and regulate differentiation [25]. 17β-Estradiol has been reported to protect rats from aldosterone-induced hypertension [51], and sex differences in the renin-angiotensin-aldosterone system have been reported [52]. Larger studies are needed to clarify the presence of heterogeneity in the association between the levels of renin-aldosterone activity and the levels of VF tissue accumulation among PA patients.

We also demonstrated that an association between the VFV/SFV ratio and eGFR was not found in the EH group. According to the findings that this relationship was augmented in the presence of high aldosterone levels, it seems reasonable that the association was found only in the PA group. However, participants diagnosed with EH in this study showed high PAC levels (153.2 [106.0, 235.5] pg/mL), which were lower than but comparable with those in the PA group. This may be because many of our patients were referred to our center because of high aldosterone levels. Although they had a higher PRA level than participants with PA, the results for the EH group in this study may not be generalizable to other populations with EH.

### Study strengths and limitations

The strengths of this study include its novelty in the assessment of the effect of the interaction between VF accumulation levels and aldosterone levels on renal function in PA.

This study has several limitations. For urinalysis, urinary albumin or 24-h urine collection is preferable, but these data were not available in this study. In addition, due to the observational nature of the study, additional studies, including an interventional and longitudinal study of weight control among patients with PA, are necessary to confirm a causal relationship.

## Conclusion

The VFV/SFV ratio was significantly associated with renal function in patients with PA, independent of the known risk factors. This association was strengthened in the presence of a high PAC. Our study suggests that control of the VFV/SFV ratio, such as by weight loss, may improve renal function, especially in male patients with high aldosterone levels. Clinicians should encourage PA patients to improve their lifestyle and reduce visceral fat tissue accumulation in addition to treating renin-aldosterone activity.

## Supplementary information

Supplementary Information
