# Supplementary material for: Association of the ratio of visceral-to-subcutaneous fat volume with renal function among patients with primary aldosteronism
Source: Hypertens Res. 2021 Aug 6;44(10):1341–51. doi: 10.1038/s41440-021-00719-w (PMC8490149; doi:10.1038/s41440-021-00719-w)
Supplement: Supplementary file 1 — Supplementary Information [file 41440_2021_719_MOESM1_ESM.doc]

This supplemental material file includes the details of methods, the supplementary tables and figures.

**Association of the ratio of visceral-to-subcutaneous fat volume with renal function among patients with primary aldosteronism**

**Authors**

Tatsuya Haze; Moe Hatakeyama; Shiro Komiya; Rina Kawano; Yuki Ohki; Shota Suzuki; Yusuke Kobayashi; Akira Fujiwara; Sanae Saka; Kouichi Tamura; Nobuhito Hirawa

**Address for correspondence**

**Nobuhito Hirawa, MD, PhD, FACP, FAHA, FJSIM, FJSH**

Department of Nephrology and Hypertension, Yokohama City University Medical Center

Address: 45-7, Urafune-cho, Minami-ku, Yokohama 232-0024, Japan.

TEL: +81-45-261-5656, FAX: +81-45-253-5713

E-mail: hirawa@yokohama-cu.ac.jp

**SUPPLEMENTAL MATERIAL TABLE OF CONTENTS**

**eMethods**

**Supplementary Table 1. Characteristics before/after imputation**

**Supplementary Table 2. Correlation between abdominal fat accumulation and renal function among patients with PA (subgroups by the presence of proteinuria using complete-case analysis)**

**Supplementary Table 3. Association between abdominal fat accumulation and eGFR (subgroup analysis by aldosterone)**

**Supplementary Table 4. Association between abdominal fat accumulation and eGFR among patients with PA (sensitivity analyses)**

**Supplementary Table 5. Association between abdominal fat accumulation and eGFR among patients with PA (subgroup analysis by sex)**

**Supplementary Table 6. Characteristics of participants with PA or EH**

**Supplementary Table 7. Correlation between abdominal fat accumulation and renin-aldosterone activity or renal function among patients with EH (n=66)**

**Supplementary Figure 1. Flowchart of participants**

**Supplementary Figure 2. Scatter plots of relation between the ratio of visceral-to-subcutaneous fat tissue volume and eGFR among patients with PA (subgroups by sex)**

**Supplementary Figure 3. Interaction between PAC and the ratio of visceral-to-subcutaneous fat tissue volume (subgroups by sex)**

**Supplementary Figure 4. Scatter plots of the relation between the ratio of visceral-to-subcutaneous fat tissue volume and eGFR among patients with EH**

**eMethods**

**Participants**

This was as a single-center cross-sectional observational study using the data from an ongoing database that were available in May 2021. Our database included 262 patients with primary aldosteronism (PA) or essential hypertension (EH) aged over 20 who underwent a confirmatory test (i.e., captopril challenge test, furosemide-upright test, or saline infusion test) between January 2006 and April 2021 at our division in Yokohama City University Medical Center (Yokohama, Japan). We excluded 16 participants who had received renal replacement therapy, who had already been treated with mineralocorticoid receptor antagonists (MRA) or adrenalectomy, or who had missing values for the estimated glomerular filtration rate (eGFR) or findings on computed tomography (CT) (**Supplementary Figure 1**).

PA was diagnosed based on the guidelines of the Japan Endocrine Society^2^ and the Japanese Society of Hypertension^14^ as (1) a high ARR (>200 with the plasma aldosterone concentration [PAC] expressed in pg/mL and plasma renin activity [PRA] expressed in ng/mL/hr) and (2) at least one positive result on a confirmatory test (i.e., captopril challenge test, furosemide-upright test, or saline infusion test).

Essential hypertension (EH) was diagnosed by each physician based on the clinical findings including the results of confirmatory tests.

**Tests for primary aldosteronism**

Abdominal CT was performed on all patients in this study. Adrenal nodules were found in 61 patients among the PA group and 13 patients among the EH group.

To determine whether aldosterone overproduction was lateralized in a unilateral adrenal gland, adrenal venous sampling (AVS) was performed in the PA group.^2,14^ Among the current study participants, 119 underwent AVS (113 with adrenocorticotropic hormone [ACTH] stimulation and 6 without ACTH) and the sampling was successful in 104. Of these 104 patients, 32 were suspected of lateralized overproduction. The success of AVS was assessed by the selectivity index (SI), which was calculated as the ratio of the plasma cortisol concentration (F) in the adrenal vein to that in the inferior vena cava. When SI was >2 without ACTH stimulation or >3 with ACTH stimulation on both sides of the adrenal veins, sampling was considered successful.^15^ The lateralized ratio (LR) was obtained by dividing the ratio of the plasma aldosterone concentration/plasma cortisol concentration (PAC/F) in the ipsilateral adrenal vein by the ratio of PAC/F in the contralateral adrenal vein. The contralateral ratio (CR) was calculated by dividing the ratio of PAC/F in the contralateral adrenal vein by the ratio of PAC/F in the inferior vena cava. Lateralization of overproduction in the adrenal glands was indicated when LR was ≥2 without ACTH stimulation,^16^ or ≥4 with ACTH stimulation or when CR was <1 with ACTH stimulation.^17^

Until the tests for PA are carried out, calcium channel blockers and alpha-blockers are preferred if possible because the other classes of antihypertensive drugs may affect the results of confirmatory tests.^2,14^

**Renal function**

The eGFR was measured every two months from 6 months before the diagnosis to the date of diagnosis (i.e., at Month -6, Month -4, Month -2, and Month 0). Not all patients attended all follow-up visits. In the PA group, the numbers of patients for which eGFR data were available were 45, 61, 120, and 158 at Month -6, Month -4, Month -2, and Month 0, respectively. The median (interquartile ranges) of each number of measurements for individuals was 2 (2, 3) times.

Quantitative urinalysis was performed using spot urine specimens by an enzymatic method for urinary creatinine and pyrogallol red-molybdate method for urinary protein. Levels under the detection limit for urinary protein were considered equivalent to 4.9 mg/dL in this study because the detection limit at our hospital was 5.0 mg/dL.

**Computed tomography**

All patients underwent multi-slice helical CT with a 0.5-5-mm slice thickness using an Aquilion TSX-101A (Canon Medical Systems Corporation, Tochigi, Japan), Aquilion ONE TSX-305A (Canon Medical Systems Corporation) or Aquilion Premium TSX-301B (Canon Medical Systems Corporation) system with a tube voltage of 120 k and automatically controlled tube current (200–500 mA), starting from the upper edge of the liver to the pelvis. Participants were examined in the supine position with both arms stretched above the head. The volume and area of visceral fat (VF) and subcutaneous fat (SF) tissue were estimated by CT using volume analyzer software (SYNAPSE VINCENT®; Fujifilm Medical Co., Tokyo) at Yokohama City University Medical Center. Two medical doctors (T.H. and M.H.) used the software. Adipose tissue was automatically detected by the software with an attenuation range between -200 and -50 Hounsfield units. The SF area was defined as adipose tissue between the skin and muscle. The VF area was defined as intra-abdominal tissue with density in the fat attenuation range.

**Assay methods for plasma aldosterone concentration and plasma renin activity**

The plasma aldosterone concentration (PAC) was measured by radioimmunoassay (SPAC-S Aldosterone Kit; Fuji Rebio, Tokyo). The reference range for PAC, measured with the patient in the supine position, was 30–159 pg/mL for both methods. Plasma renin activity (PRA) was measured by radioimmunoassay. The normal reference range for PRA, measured with the patient in the supine position, was 0.3–2.9 ng/mL per hr (PRA-FR radioimmunoassay kits; Fuji Rebio). PAC and PRA were measured in the supine position after at least 30-min of rest in the morning in the fasted state.

**Other measurements**

In-clinic blood pressure (BP) was measured by medical staff using a standard sphygmomanometer or an automated device after at least 30-min of rest in a seated position. Measurement was carried out two times with intervals in one clinic visit. When the two measurements differed significantly, an additional measurement was performed. The mean value of the two measurements with stable values was obtained.^14^ The mean arterial pressure was calculated using a formula in which the diastolic BP (DBP) is doubled and added to the systolic BP (SBP), and then divided by 3.

The diagnostic criteria for diabetes and dyslipidemia followed the recommendations from the Japan Diabetes Society and the Japan Atherosclerosis Society.^20,21^

**Sensitivity analyses**

We conducted eleven sensitivity analyses and subgroup analyses. First, we used the VF tissue volume per body weight, SF tissue volume per body weight, VF tissue area per height, and SF area per height instead of the VF tissue volume, SF tissue volume, VF tissue area, and SF area, respectively. Second, we adjusted for the covariates included in Model 4 (see main text), but using the log-transformed PRA instead of PAC. Third, we adjusted for the covariates included in Model 4, but using the log-transformed aldosterone-to-renin ratio (ARR) instead of PAC. Fourth, we adjusted for the covariates included in Model 4 and the use of angiotensin-converting enzyme inhibitors (ACEi) or angiotensin receptor blockers (ARB). Fifth, we adjusted for the covariates included in Model 4, but using hemoglobin A1c, low-density lipoprotein cholesterol, high-density lipoprotein cholesterol, and log-transformed triglyceride instead of history of diabetes and the prevalence of dyslipidemia. Sixth, we adjusted for the covariates included in Model 4 and the number of measurements of eGFR. Seventh, we adjusted for the covariates included in Model 4 and the total psoas muscle volume. Eighth, we performed subgroup analyses between participants with lateralization of the overproduction of aldosterone in the adrenal glands in AVS (*n*=32) and those without (*n*=72). Ninth, we performed subgroup analyses between participants who had adenomatous adrenal glands on CT (*n*=61) and those who did not (*n*=119). Tenth, we excluded participants who were taking ACEi, ARB, diuretics, beta-blockers, or thiazolidines which were reported to affect PAC or the VFV/SFV ratio.^26,27^ Eleventh, we performed complete-case analyses without imputation (*n*=145).

**Subgroup analyses by sex**

We divided the PA group into the female group (*n*=108) and the male group (*n*=72) and assessed the linear regression models and the interactions for PAC which were obtained in the overall analyses. Scatter plots of the relation between eGFR and the VFV/SFV ratio in these subgroups are shown in **Supplementary Figure 2**. Scatter plots of the interaction between PAC and the VFV/SFV ratio are shown in **Supplementary Figure 3**.

| **Supplementary Table 1. Characteristics before/after imputation** | | | |
| --- | --- | --- | --- |
| **Characteristics** | **Before imputation** | **Missing, n (%)** | **After imputation**  **(*n*=246)** |
| Age, years | 52.8±11.6 | 0 (0.0) | 52.8±11.6 |
| Women, n (%) | 133 (54.1) | 0 (0.0) | 133 (54.1) |
| BMI, kg/m^2^ | 25.1±4.5 | 0 (0.0) | 25.1±4.5 |
| History of diabetes, n (%) | 22 (8.9) | 0 (0.0) | 22 (8.9) |
| Current smokers, n (%) | 55 (22.4) | 0 (0.0) | 55 (22.4) |
| History of cardiovascular disease, n (%) | 15 (6.1) | 0 (0.0) | 15 (6.1) |
| Known duration of hypertension, years | 4.0 (1.2, 10.0) | 3 (1.2) | 4.0 (1.3, 10.0) |
| Antihypertensive medication use, n (%) | 84 (34.1) | 0 (0.0) | 84 (34.1) |
| Calcium-channel blockers, n (%) | 74 (30.1) | 0 (0.0) | 74 (30.1) |
| Angiotensin-converting enzyme inhibitors or  Angiotensin receptor blockers, n (%) | 15 (6.1) | 0 (0.0) | 15 (6.1) |
| Thiazides, n (%) | 7 (2.8) | 0 (0.0) | 7 (2.8) |
| Alpha-blockers, n (%) | 15 (6.1) | 0 (0.0) | 15 (6.1) |
| Beta-blockers, n (%) | 4 (1.6) | 0 (0.0) | 4 (1.6) |
| SBP, mmHg | 147±19 | 1 (0.4) | 147±19 |
| DBP, mmHg | 92±11 | 1 (0.4) | 92±11 |
| PAC, pg/mL | 168.0 (125.2, 234.9) | 0 (0.0) | 168.0 (125.2, 234.9) |
| PRA, ng/mL per hour | 0.5 (0.3, 0.8) | 0 (0.0) | 0.5 (0.3, 0.8) |
| ARR (=PAC/PRA) | 337.5 (224.2, 556.5) | 0 (0.0) | 337.5 (224.2, 556.5) |
| eGFR, mL/min/1.73 m^2^ | 76.4±16.0 | 0 (0.0) | 76.4±16.0 |
| Urinary protein, mg/gCr | 96.3 (57.6, 186.5) | 43 (17.5) | 98.6 (60.4, 252.6) |
| Serum potassium, mEq/L | 4.0±0.4 | 0 (0.0) | 4.0±0.4 |
| Uric acid, mg/dL | 5.6±1.3 | 11 (4.5) | 5.6±1.4 |
| HbA1c, % | 5.5 (5.3, 5.9) | 59 (24.0) | 5.6 (5.3, 6.2) |
| Total cholesterol, mg/dL | 202.4±34.3 | 90 (36.6) | 205.8±40.6 |
| Triglycerides, mg/dL | 111.8 (77.4, 162.2) | 18 (7.3) | 113.3 (76.4, 177.3) |
| LDL cholesterol, mg/dL | 118.3±28.4 | 21 (8.5) | 119.3±30.3 |
| HDL cholesterol, mg/dL | 58.7±15.8 | 44 (17.9) | 59.1±17.1 |
| WC, cm | 87.9±10.7 | 0 (0.0) | 87.9±10.7 |
| VFA, cm^2^ | 120.6±65.3 | 0 (0.0) | 120.6±65.3 |
| SFA, cm^2^ | 167.8±82.9 | 0 (0.0) | 167.8±82.9 |
| VFA/SFA ratio | 0.8±0.6 | 0 (0.0) | 0.8±0.6 |
| VFV, cm^3^ | 3208.3±1881.3 | 0 (0.0) | 3208.3±1881.3 |
| SFV, cm^3^ | 4411.7±2219.4 | 0 (0.0) | 4411.7±2219.4 |
| VFV/SFV ratio | 0.8±0.4 | 0 (0.0) | 0.8±0.4 |
| Total psoas muscle volume, cm^3^ | 319.3±124.0 | 0 (0.0) | 319.3±124.0 |
| All variables with missing data were imputed with 100 data sets using chained equations using an entire data set. Data are expressed as the mean±SD for unskewed variables and median (interquartile range) for skewed variables. ARR=aldosterone-to-renin ratio; BMI=body mass index; DBP=diastolic blood pressure; eGFR=estimated glomerular filtration rate; gCr=per gram of creatinine; HDL=high-density lipoprotein; LDL=low-density lipoprotein; PAC=plasma aldosterone concentration; PRA=plasma renin activity; SBP=systolic blood pressure; SD=standard deviation; SFA=subcutaneous fat area; SFV=subcutaneous fat volume; HbA1c=hemoglobin A1c; VFA=visceral fat area; VFV=visceral fat volume; WC=waist circumference. | | | |

| **Supplementary Table 2. Correlation between abdominal fat accumulation and renal function among patients with PA (subgroups by the presence of proteinuria using complete-case analysis)** | | | | | | |
| --- | --- | --- | --- | --- | --- | --- |
| **Estimated Pearson's product-moment correlation coefficients (*r*)** | | | | | | |
| **Participants without proteinuria (*n*=106)** | | | | | | |
|  | **VFV, cm³** | **SFV, cm³** | **VFV/SFV ratio** | **VFA, cm^2^** | **SFA, cm^2^** | **VFA/SFA ratio** |
| eGFR, mL/min/1.73 m^2^ | -0.27† (-0.46, -0.08) | 0.22* (0.05, 0.39) | -0.43‡ (-0.60, -0.26) | -0.20* (-0.39, -0.01) | 0.21* (0.04, 0.39) | -0.37‡ (-0.55, -0.20) |
| log Urinary protein, mg/gCr | -0.02 (-0.21, 0.17) | 0.09 (-0.10, 0.28) | -0.13 (-0.32, 0.06) | -0.05 (-0.24, 0.15) | 0.05 (-0.14, 0.24) | -0.12 (-0.31, 0.08) |
| **Participants with proteinuria (*n*=39)** | | | | | | |
|  | **VFV, cm³** | **SFV, cm³** | **VFV/SFV ratio** | **VFA, cm^2^** | **SFA, cm^2^** | **VFA/SFA ratio** |
| eGFR, mL/min/1.73 m^2^ | -0.45† (-0.75, -0.16) | 0.06 (-0.24, 0.37) | -0.70‡ (-0.90, -0.49) | -0.34* (-0.65, -0.03) | 0.08 (-0.22, 0.39) | -0.50† (-0.78, -0.22) |
| log Urinary protein, mg/gCr | 0.61‡ (0.44, 0.77) | 0.15 (-0.15, 0.44) | 0.63‡ (0.46, 0.79) | 0.52‡ (0.32, 0.71) | 0.04 (-0.27, 0.35) | 0.55‡ (0.37, 0.74) |
| Estimated Pearson's product-moment correlation coefficients (95% confidence intervals) are shown. 145 participants without missing values for urinary protein were divided into two groups by the presence of proteinuria (i.e., ≥150 mg/gCr). To improve normality, urinary protein was log-transformed. ARR=aldosterone-to-renin ratio; eGFR=estimated glomerular filtration rate; gCr=per gram of creatinine; PA=primary aldosteronism; PAC=plasma aldosterone concentration; PRA=plasma renin activity; SFA=subcutaneous fat area; SFV=subcutaneous fat volume; VFA=visceral fat area; VFV=visceral fat volume. **p*<0.05; †*p*<0.01; ‡*p*<0.001. | | | | | | |

| **Supplementary Table 3. Association between abdominal fat accumulation and eGFR (subgroup analysis by aldosterone)** | | | | | | |
| --- | --- | --- | --- | --- | --- | --- |
| **Estimates for eGFR per each 1-SD higher** | | | | | | |
|  | **VFV, cm³** | **SFV, cm³** | **VFV/SFV ratio** | **VFA, cm^2^** | **SFA, cm^2^** | **VFA/SFA ratio** |
| **The lower PAC group (*n*=90)** | | | | | | |
| **Model 1** | -1.67 (-4.08, 0.74) | 1.43 (-0.98, 3.85) | -3.02* (-5.37, -0.67) | -0.58 (-3.01, 1.85) | 1.05 (-1.38, 3.47) | -2.29 (-4.67, 0.10) |
| **Model 2** | 0.52 (-2.67, 3.71) | 2.27 (-1.11, 5.65) | -0.47 (-3.94, 3.00) | 2.46 (-0.36, 5.27) | 2.04 (-1.28, 5.36) | 0.05 (-2.74, 2.84) |
| **Model 3** | 0.68 (-2.65, 4.00) | 2.87 (-0.51, 6.24) | -1.01 (-4.44, 2.42) | 1.97 (-1.01, 4.95) | 2.74 (-0.62, 6.10) | -1.19 (-4.17, 1.78) |
| **Model 4** | 0.05 (-3.30, 3.40) | 3.16 (-0.19, 6.51) | -1.58 (-4.93, 1.78) | 0.83 (-2.26, 3.93) | 2.75 (-0.55, 6.05) | -2.09 (-5.01, 0.82) |
| **The higher PAC group (*n*=90)** | | | | | | |
| **Model 1** | -8.25‡ (-11.47, -5.04) | 1.45 (-2.19, 5.08) | -11.18‡ (-13.99, -8.38) | -6.39‡ (-9.79, -3.00) | 1.88 (-1.75, 5.51) | -4.14* (-7.69, -0.60) |
| **Model 2** | -5.37* (-9.68, -1.05) | 2.30 (-3.11, 7.70) | -7.98‡ (-11.84, -4.12) | -0.38 (-4.37, 3.61) | 2.22 (-2.75, 7.20) | -0.58 (-3.32, 2.16) |
| **Model 3** | -5.24* (-9.96, -0.52) | 1.68 (-4.02, 7.38) | -7.88‡ (-11.84, -3.92) | 0.51 (-3.85, 4.86) | 1.56 (-3.54, 6.67) | 0.13 (-2.66, 2.92) |
| **Model 4** | -4.32 (-8.66, 0.03) | 3.40 (-1.87, 8.67) | -7.66‡ (-11.25, -4.06) | -0.70 (-4.75, 3.35) | 3.50 (-1.21, 8.21) | -0.43 (-3.14, 2.27) |
| We divided participants in the PA group into two groups according to the median PAC value (i.e., PAC≤175.0 pg/mL and PAC>175.0 pg/mL). Unadjusted and adjusted estimates (95% confidence intervals) for eGFR associated with one-SD higher VFV, SFV, the VFV/SFV ratio, VFA, SFA, and the VFA/SFA ratio are shown. Each one-SD increment is as follows: For the higher PAC subgroup, VFV, 2036.4 cm³; SFV, 2303.8 cm³; the VFV/SFV ratio, 0.5; VFA, 67.3 cm²; SFA, 84.3 cm²; the VFA/SFA ratio, 0.8. For the lower PAC subgroup, VFV, 1719.8 cm³ ; SFV, 2198.3 cm³; the VFV/SFV ratio, 0.4; VFA, 64.4 cm²; SFA, 84.1 cm²; the VFA/SFA ratio, 0.4. Model 1 was unadjusted. Model 2 was adjusted for age, sex, BMI, and laterality of aldosterone hypersecretion. Model 3 was adjusted for the covariates included in Model 2 and history of diabetes, prevalence of dyslipidemia, current smoking status, and mean artery pressure. Model 4 was adjusted for the covariates included in Model 3 and serum potassium, log-transformed duration of hypertension, log-transformed urinary protein, and log-transformed PAC. Exposures were included in models separately. BMI=body mass index; eGFR=estimated glomerular filtration rate; PA=primary aldosteronism; PAC=plasma aldosterone concentration; SD=standard deviation; SFA=subcutaneous fat area; SFV=subcutaneous fat volume; VFA=visceral fat area; VFV=visceral fat volume. **p*<0.05; †*p*<0.01; ‡*p*<0.001. | | | | | | |

| **Supplementary Table 4. Association between abdominal fat accumulation and eGFR among patients with PA (sensitivity analyses)** | | |
| --- | --- | --- |
| **Estimates for eGFR per each 1-SD higher** | | |
| **Models** | | **VFV/SFV ratio** |
| **Model 4** | **Same as Model 4 in Table 3** | -4.56‡ (-6.98, -2.14) |
| **Model 5** | **Adjusted for PRA** | -4.48‡ (-6.99, -1.97) |
| **Model 6** | **Adjusted for ARR** | -4.78‡ (-7.28, -2.28) |
| **Model 7** | **Adjusted for RASi use** | -4.57‡ (-7.00, -2.15) |
| **Model 8** | **Adjusted for psoas muscle volume** | -4.17† (-6.66, -1.68) |
| **Model 9** | **Excluding participants treated with RASi, beta-blockers, diuretics, or thiazolidines (*n=*167)** | -4.87‡ (-7.28, -2.47) |
| **Model 10** | **Complete-case analysis (*n*=145)** | -4.56‡ (-7.18, -1.94) |
| Unadjusted and adjusted estimates (95% confidence intervals) for eGFR associated with one-SD higher the VFV/SFV ratio, and the VFA/SFA ratio are shown. Model 4 was adjusted for age, sex, BMI, laterality of aldosterone hypersecretion, history of diabetes, prevalence of dyslipidemia, current smoking status, mean artery pressure, serum potassium, log-transformed duration of hypertension, log-transformed urinary protein, and log-transformed PAC. Model 5 was adjusted for log-transformed PRA instead of PAC. Model 6 was adjusted for log-transformed ARR instead of PAC. Model 7 was adjusted for the covariates included in Model 4 and RASi use (yes or no). Model 8 was adjusted for the covariates included in Model 4 and the total psoas muscle volume. Model 9 excluded participants treated with RASi, beta-blockers, diuretics, or thiazolidines and was adjusted for the covariates in Model 4 (*n*=167). Model 10 was the complete-case analysis using the covariates in Model 4 (*n*=145). Exposures were included in models separately. ARR=aldosterone-to-renin ratio; BMI=body mass index; eGFR=estimated glomerular filtration rate; PA=primary aldosteronism; PAC=plasma aldosterone concentration; PRA=plasma renin activity; RASi=angiotensin-converting enzyme inhibitors or angiotensin receptor blockers; SD=standard deviation; SFA=subcutaneous fat area; SFV=subcutaneous fat volume; VFA=visceral fat area; VFV=visceral fat volume. **p*<0.05; †*p*<0.01; ‡*p*<0.001. | | |

| **Supplementary Table 5. Association between abdominal fat accumulation and eGFR among patients with PA (subgroup analysis by sex)** | | |
| --- | --- | --- |
| **Estimates for eGFR per each 1-SD higher** | | |
|  | **VFV/SFV ratio** | |
|  | **Women (*n*=108)** | **Men (*n*=72)** |
| **Model 1** | -5.64‡ (-7.79, -3.48) | -7.17‡ (-10.62, -3.73) |
| **Model 4** | -2.49* (-4.88, -0.10) | -4.05† (-6.64, -1.47) |
| We divided participants in the PA group into two groups according to sex. Unadjusted and adjusted estimates (95% confidence intervals) for eGFR associated with one-SD higher the VFV/SFV ratio are shown. Each one-SD increment is as follows: For the female subgroup, the VFV/SFV ratio, 0.2. For the male subgroup, the VFV/SFV ratio, 0.4. Model 1 was unadjusted. Model 4 was same as in Table 3 and was adjusted for age, sex, BMI, laterality of aldosterone hypersecretion, history of diabetes, prevalence of dyslipidemia, current smoking status, mean artery pressure, serum potassium, log-transformed duration of hypertension, log-transformed urinary protein, and log-transformed PAC. BMI=body mass index; eGFR=estimated glomerular filtration rate; PA=primary aldosteronism; PAC=plasma aldosterone concentration; SD=standard deviation; SFV=subcutaneous fat volume; VFV=visceral fat volume. **p*<0.05; †*p*<0.01; ‡*p*<0.001. | | |

| **Supplementary Table 6. Characteristics of participants with PA or EH** | | | |
| --- | --- | --- | --- |
| **Characteristics** | **PA group**  **(*n*=180)** | **EH group**  **(*n*=66)** | ***p*-value** |
| Age, years | 52.7±11.0 | 53.0±13.2 | 0.85 |
| Women, n (%) | 108 (60.0) | 25 (37.9) | <0.01† |
| BMI, kg/m^2^ | 25.1±4.7 | 25.1±4.2 | 0.92 |
| History of diabetes, n (%) | 18 (10.0) | 4 (6.1) | 0.34 |
| Current smokers, n (%) | 34 (18.9) | 21 (31.8) | <0.05 |
| History of cardiovascular disease, n (%) | 11 (6.1) | 4 (6.1) | 0.99 |
| Known duration of hypertension, years | 3.6 (1.2, 9.0) | 5.5 (1.7, 15.5) | 0.06 |
| Antihypertensive medication use, n (%) | 71 (39.4) | 13 (19.7) | <0.01† |
| Calcium-channel blockers, n (%) | 68 (37.8) | 6 (9.1) | <0.001‡ |
| Angiotensin-converting enzyme inhibitors or Angiotensin receptor blockers, n (%) | 10 (5.6) | 5 (7.6) | 0.56 |
| Thiazides, n (%) | 4 (2.2) | 3 (4.5) | 0.33 |
| Alpha-blockers, n (%) | 12 (6.7) | 3 (4.5) | 0.54 |
| Beta-blockers, n (%) | 3 (1.7) | 1 (1.5) | 0.93 |
| SBP, mmHg | 144±16 | 157±24 | <0.001‡ |
| DBP, mmHg | 90±10 | 96±14 | <0.001‡ |
| PAC, pg/mL | 175.0 (129.0, 234.6) | 153.2 (106.0, 235.5) | 0.21 |
| PRA, ng/mL per hour | 0.4 (0.2, 0.6) | 1.0 (0.5, 1.7) | <0.001‡ |
| ARR (=PAC/PRA) | 432.5 (294.9, 670.7) | 207.2 (108.9, 255.0) | <0.001‡ |
| eGFR, mL/min/1.73 m^2^ | 77.5±14.8 | 73.3±18.7 | 0.06 |
| Urinary protein, mg/gCr | 97.7 (59.0, 278.9) | 105.2 (64.8, 226.4) | 0.68 |
| Serum potassium, mEq/L | 3.9±0.4 | 4.1±0.4 | <0.01† |
| Uric acid, mg/dL | 5.4±1.3 | 6.2±1.4 | <0.001‡ |
| HbA1c, % | 5.6 (5.3, 6.2) | 5.5 (5.3, 6.0) | 0.10 |
| Total cholesterol, mg/dL | 206.6±40.3 | 203.7±41.3 | 0.66 |
| Triglycerides, mg/dL | 115.7 (79.9, 185.2) | 106.9 (68.4, 163.9) | 0.21 |
| LDL cholesterol, mg/L | 119.3±29.0 | 119.2±33.7 | 0.97 |
| HDL cholesterol, mg/L | 59.6±17.2 | 58.0±16.9 | 0.65 |
| WC, cm | 88.0±10.6 | 87.7±11.2 | 0.87 |
| VFA, cm^2^ | 118.3±65.9 | 127.1±63.9 | 0.35 |
| SFA, cm^2^ | 171.4±84.0 | 158.0±79.6 | 0.26 |
| VFA/SFA ratio | 0.8±0.6 | 0.9±0.4 | 0.38 |
| VFV, cm^3^ | 3139.2±1884.9 | 3396.7±1872.8 | 0.34 |
| SFV, cm^3^ | 4484.6±2245.4 | 4212.8±2151.2 | 0.40 |
| VFV/SFV ratio | 0.8±0.4 | 0.8±0.4 | 0.19 |
| Total psoas muscle volume, cm^3^ | 304.7±113.0 | 359.1±143.7 | <0.01† |
| Data are expressed as the mean±SD for unskewed variables and median (interquartile range) for skewed variables. ARR=aldosterone-to-renin ratio; BMI=body mass index; DBP=diastolic blood pressure; eGFR=estimated glomerular filtration rate; EH=essential hypertension; gCr=per gram of creatinine; HDL=high-density lipoprotein; LDL=low-density lipoprotein; PA=primary aldosteronism; PAC=plasma aldosterone concentration; PRA=plasma renin activity; SBP=systolic blood pressure; SD=standard deviation; SFA=subcutaneous fat area; SFV=subcutaneous fat volume; HbA1c=hemoglobin A1c; HDL=high-density lipoprotein; VFA=visceral fat area; VFV=visceral fat volume; WC=waist circumference. Comparison tests were performed between two groups with the Student’s t-test, Wilcoxon rank-sum test, or chi-square test where appropriate. **p*<0.05; †*p*<0.01; ‡*p*<0.001. | | | |

| **Supplementary Table 7. Correlation between abdominal fat accumulation and renin-aldosterone activity or renal function among patients with EH (*n*=66)** | | | | | | |
| --- | --- | --- | --- | --- | --- | --- |
| **Estimated Pearson's product-moment correlation coefficients (*r*)** | | | | | | |
| **Renin-aldosterone activity** | | | | | | |
|  | **VFV, cm³** | **SFV, cm³** | **VFV/SFV ratio** | **VFA, cm^2^** | **SFA, cm^2^** | **VFA/SFA ratio** |
| log PAC,  pg/mL | 0.02 (-0.22, 0.26) | 0.07 (-0.17, 0.31) | -0.07 (-0.31, 0.17) | 0.00 (-0.24, 0.24) | 0.04 (-0.20, 0.28) | -0.02 (-0.26, 0.23) |
| log PRA, ng/mL/hr | 0.03 (-0.21, 0.27) | -0.01 (-0.26, 0.23) | 0.04 (-0.21, 0.28) | 0.06 (-0.18, 0.30) | -0.00 (-0.25, 0.24) | 0.06 (-0.19, 0.30) |
| log ARR | -0.02 (-0.26, 0.22) | 0.06 (-0.19, 0.30) | -0.08 (-0.32, 0.16) | -0.07 (-0.31, 0.17) | 0.03 (-0.21, 0.27) | -0.07 (-0.31, 0.17) |
| **Renal function** | | | | | | |
|  | **VFV, cm³** | **SFV, cm³** | **VFV/SFV ratio** | **VFA, cm^2^** | **SFA, cm^2^** | **VFA/SFA ratio** |
| eGFR, mL/min/1.73 m^2^ | 0.03 (-0.21, 0.27) | 0.04 (-0.20, 0.28) | 0.00 (-0.24, 0.24) | 0.14 (-0.10, 0.37) | 0.02 (-0.22, 0.26) | 0.15 (-0.09, 0.38) |
| log Urinary protein, mg/gCr | 0.05 (-0.28, 0.37) | 0.11 (-0.18, 0.39) | -0.05 (-0.30, 0.21) | -0.03 (-0.35, 0.30) | 0.03 (-0.27, 0.32) | -0.06 (-0.31, 0.19) |
| Estimated Pearson's product-moment correlation coefficients (95% confidence intervals) among patients with essential hypertension are shown. To improve normality, PAC, PRA, ARR, and urinary protein were log-transformed. ARR=aldosterone-to-renin ratio; eGFR=estimated glomerular filtration rate; EH=essential hypertension; gCr=per gram of creatinine; PAC=plasma aldosterone concentration; PRA=plasma renin activity; SFA=subcutaneous fat area; SFV=subcutaneous fat volume; VFA=visceral fat area; VFV=visceral fat volume. **p*<0.05; †*p*<0.01; ‡*p*<0.001. | | | | | | |

**Supplementary Figure 1. Flowchart of participants**

The flowchart of participants included in the current study is shown. Primary analyses were conducted using a dataset of patients with PA (*n*=180). CT=computed tomography; eGFR=estimated glomerular filtration rate; MRA=mineralocorticoid receptor antagonists; PA=primary aldosteronism; PAC=plasma aldosterone concentration; EH=essential hypertension.

**Supplementary Figure 2. Scatter plots of the relation between the ratio of visceral-to-subcutaneous fat tissue volume and eGFR among patients with PA (subgroups by sex)**

Scatter plots of the relation between the VFV/SFV ratio and eGFR among patients with primary aldosteronism are shown. Each dot represents a value observed in an individual patient. Circular dots (green) represent data for the male group (*n*=72). Triangular dots (orange) represent data for the female group (*n*=108). The lines represent simple linear regression models. The *p*-values were calculated for the Pearson's product-moment correlation coefficients (*r*-values). eGFR=estimated glomerular filtration rate; PA=primary aldosteronism; SFV=subcutaneous fat volume; VFV=visceral fat volume.

**Supplementary Figure 3. Interaction between PAC and the ratio of visceral-to-subcutaneous fat tissue volume (subgroups by sex)**

Scatter plots of the relation between eGFR and the VFV/SFV ratio are shown for (**A**) the female PA group and (**B**) the male PA group. Each dot represents a value observed value in an individual patient. Circular dots (red) represent data for the group with higher PAC levels (PAC>175.0 pg/mL). Triangular dots (blue) represent data for the group with lower PAC levels (PAC≤175.0 pg/mL). The red lines represent the simple regression model for the higher PAC group, respectively. The blue lines represent the simple regression model for the lower PAC group, respectively. The *p*-value was calculated for the multiple interaction terms between the VFV/SFV ratio and higher PAC vs. lower PAC group in the regression models. eGFR=estimated glomerular filtration rate; PA=primary aldosteronism; PAC=plasma aldosterone concentration; SFV=subcutaneous fat volume; VFV=visceral fat volume.

**Supplementary Figure 4. Scatter plots of the relation between the ratio of visceral-to-subcutaneous fat tissue volume and eGFR among patients with EH**

Scatter plots of the relation between eGFR and (A) VFV, (B) SFV, (C) the VFV/SFV ratio, and (D) the sum of VFV and SFV among patients with EH are shown. Each black dot represents a value observed value in an individual patient. The black lines represent simple linear regression models. The p-values were calculated for the Pearson's product-moment correlation coefficients (*r-*values). eGFR=estimated glomerular filtration rate; EH=essential hypertension; n.s.=not significant; SFV=subcutaneous fat volume; VFV=visceral fat volume.
